# Supplementary material for: Auditory beat perception is related to speech output fluency in post-stroke aphasia
Source: Sci Rep. 2021 Feb 4;11:3168. doi: 10.1038/s41598-021-82809-w (PMC7862238; doi:10.1038/s41598-021-82809-w)
Supplement: Supplementary file 2 — Supplementary Information 2. [file 41598_2021_82809_MOESM2_ESM.docx]

**Auditory beat perception is related to speech output fluency in post-stroke aphasia**

James D. Stefaniak^1,2^, Matthew A. Lambon Ralph^2^, Blanca De Dios Perez^3^, Timothy D. Griffiths^4,5*^, Manon Grube^4,6*^

^1^Division of Neuroscience and Experimental Psychology, University of Manchester, Manchester Academic Health Science Centre, Oxford Road, Manchester, UK

^2^MRC Cognition and Brain Sciences Unit, University of Cambridge, Cambridge, UK

^3^Division of Psychiatry and Applied Psychology, University of Nottingham, Nottingham, UK

^4^Newcastle University Medical School, Framlington Place, Newcastle-upon-Tyne, UK

^5^Wellcome Centre for Human Neuroimaging, University College London, UK

^6^Center for Music in the Brain, Department of Clinical Medicine, Aarhus University, Denmark

*These authors contributed equally to this work.

Correspondence to: Dr James Stefaniak, MRC Cognition and Brain Sciences Unit, University of Cambridge, Cambridge, UK. E-mail: james.stefaniak@cantab.net

**Supplementary Methods**

**Participants**

Participants in the entire cohort of 76 stroke survivors were native English speakers with normal or corrected-to-normal vision and/or hearing who were at least one year post left-hemispheric stroke (either ischaemic or haemorrhagic). Exclusion criteria included: more than one stroke; other neurological condition; severe motor-speech disorder; visual neglect; any contraindications for MRI scanning; or being pre-morbidly left handed. Aphasia was diagnosed and classified using the BDAE^1^. Participants with PSA were recruited regardless of aphasia severity or classification, in order to sample the full range of aphasia symptoms. The 17 participants in the current PSA subgroup were recruited from the larger cohort of 76 stroke survivors on the basis of willingness and availability to undergo further psychoacoustic testing.

**Lesion overlap map**

Structural T_1_-weighted MRI scans were acquired on a 3.0 T Philips Achieva scanner (Philips Healthcare) using an 8-element SENSE head coil. A T_1_-weighted inversion recovery sequence with 3D acquisition was used with the following parameters: repetition time = 9.0ms, echo time = 3.93ms, flip angle = 8°, 150 contiguous slices, slice thickness = 1mm, acquired voxel size = 1.0 x 1.0 x 1.0mm, matrix size 256 x 256, field of view = 256mm, inversion time = 1150ms, SENSE acceleration factor 2.5, total scan acquisition time = 575s. The lesion overlap map was generated using FSL’s ‘fslmaths’ command^2^ and displayed in MRIcron ^3^ (www.mricro.com/mricron/).

**Neuropsychological tests**

The entire cohort of 76 stroke survivors had previously been administered an extensive battery of neuropsychological tests and principal component analysis (PCA) results from this database have been published from earlier data collection rounds^4,5^. We expected immediate non-word and word repetition (subtests 8 and 9 from the Psycholinguistic Assessment of Language Processing in Aphasia battery^6^), Boston Naming Test (BNT)^7^, Forward Digit Span^8^, and Cambridge Semantic Battery picture naming^9^ to represent participants’ phonological ability. We expected the Cambridge Semantic Battery spoken word-to-picture matching^9^, spoken sentence comprehension from the Comprehension Aphasia Test (CAT)^10^, 96-trial synonym judgement test^11^ and Type-Token Ratio from the ‘Cookie theft’ description (Boston Diagnostic Aphasia Examination^1^) to represent participants’ semantic ability. We expected the number of speech tokens, Mean Length of Utterance, and Words Per Minute from the ‘Cookie theft’ description (Boston Diagnostic Aphasia Examination^1^) to represent participants’ speech fluency. We expected the Raven’s Coloured Progressive Matrices^12^ and the Brixton Spatial Anticipation Test^13^ to represent participants’ executive function.

The ‘Cookie theft’ description from the Boston Diagnostic Aphasia Examination^1^ involved being shown the ‘Cookie theft’ picture and being asked ‘tell me everything you see going on in this picture’. Responses were recorded, transcribed and used to obtain various parameters of connected speech production (Type-Token Ratio, number of speech tokens, Mean Length of Utterance, Words Per Minute) using the coding method described previously^4^.

**Psychoacoustic tests of pitch, rhythm and timbre**

Psychoacoustic testing occurred over several sessions for each participant with PSA, with the number of sessions determined by the participant so as to avoid fatigue impacting on performance. With the controls, psychoacoustic testing was always completed within a single session. Due to time constraints, one control was not tested on P2 or P3, and a second healthy control was not tested on P1.

We define tone sequences as more than two tones grouped together. Thus, four of the seven psychoacoustic tests required processing of tone sequences: P2 (pitch local change detection); P3 (pitch global change detection); R2 (isochrony deviation detection); and R3 (metrical pattern discrimination). Three of the seven psychoacoustic tests did not require processing of tone sequences: P1 (pitch basic change detection); R1 (single time interval discrimination); and DM (Dynamic Modulation detection).

**Statistical analysis**

All variables were assessed for normality using the Kolmogorov-Smirnov test.

Group differences between PSA and control participants were initially assessed using the independent samples *t*-test for age (normally distributed), Pearson’s Chi-square test for sex, and Mann-Whitney U test for years of education (not normally distributed; p=0.00002 in PSA). As several pure tone audiometry thresholds were not normally distributed, group differences of audiometry thresholds between PSA and control participants were assessed using Mann-Whitney U tests.

As the PSA subgroup and control group had significantly different years of education (see Results), and years of education was not normally distributed, group differences between PSA and control participants on neuropsychological and psychoacoustic measures were assessed using a non-parametric one-way rank analysis of covariance (ANCOVA) with years of education included as the covariate^14^.

It was possible that participants with PSA might have been significantly impaired on psychoacoustic tasks at the individual level, even if there was no group difference compared to controls. We therefore compared, individually, the psychoacoustic scores of each participant with PSA to the control group using the Bayesian Test for a Deficit controlling for years of education as a covariate (https://homepages.abdn.ac.uk/j.crawford/pages/dept/SingleCaseMethodsComputerPrograms.HTM)^15^. This method provides a Bayesian point estimate of the proportion of controls expected to perform worse than the individual case, which corresponds to the one-sided p-value for a significance test of whether the case’s score can be assumed to equal an observation from the control population^16^. Since this method assumes a Gaussian distribution, psychoacoustic variables that were not normally distributed in either the PSA or control group (P1, P3, R1, R2, DM) were log_10_ transformed. If the log_10_ transformed variable was not normally distributed, alternative transformations were used. R2 only became normally distributed in controls after square root transformation. P3 only became normally distributed in controls after quartic transformation. P1 remained non-normally distributed in the control group, no matter the transformation applied (p=0.01 for log_10_P1).

Given the large number of neuropsychological measures available for our PSA subgroup and the whole cohort we recruited them from, we used a varimax-rotated PCA to reduce these scores to a smaller number of dimensions, as has been done previously^4,5^. As PCA was unlikely to be stable in our subgroup of 17 PSA participants with psychoacoustic data^17^, we performed the PCA on the correlation matrix of neuropsychological test scores of the entire cohort of PSA participants (n=76), which has been shown formally to be highly reliable and stable. Scores from Principal Components (PCs) with an eigenvalue greater than 1 were taken to be estimates of underlying cognitive components in our 17 PSA participants and were used in correlation analyses with the psychoacoustic measures.

Within the PSA subgroup, correlations were performed between neuropsychological test scores, neuropsychological ‘PC’ scores and psychoacoustic scores. Non-parametric Spearman correlations were performed because several variables (P1, R2, DM, PC1) were not normally distributed.

We used SPSS version 25 for the above statistical analyses, except for partial Spearman correlations and rank ANCOVAs, which were performed in Matlab R2018a. We defined statistical significance as p<0.05 with Bonferroni correction (by the number of tests) applied to the significance thresholds (i.e. reported p-values are uncorrected). Reported p-values for correlations between neuropsychological and/or psychoacoustic scores are one-tailed due to *a priori* hypotheses as to the direction of the associations, i.e. that better performance on auditory tasks would be associated with higher language scores.

**Supplementary results**

**Supplementary Table S1: Demographic, neuropsychological and psychoacoustic variables in participants with post-stroke aphasia and controls**

| **Identifier** |  | | | **Neuropsychological measures** | | | | **Psychoacoustic measures** | | | | | | |
| --- | --- | --- | --- | --- | --- | --- | --- | --- | --- | --- | --- | --- | --- | --- |
|  | **Age** | **Sex** | **Years education** | **Raven’s (36)** | **Words Per Minute** | **Mean Length of Utterance** | **Speech Tokens** | **P1** | **P2** | **P3** | **R1** | **R2** | **R3** | **DM** |
| **Healthy controls** |  |  |  |  |  |  |  |  |  |  |  |  |  |  |
| AB | 65 | F | 16 | 29 | 111.4 | 11.2 | 98 | 0.33 | 100 | 98 | 39.0 | 32.0 | 18.0 | 0.13 |
| ACJ | 62 | M | 17 | 34 | 159.0 | 25.0 | 103 | 0.27 | 98 | 98 | 11.0 | 0.7 | 13.0 | 0.14 |
| AT | 65 | M | 20 | 36 | 130.2 | 20.5 | 106 | 0.20 | 93 | 98 | 14.0 | 6.3 | 7.3 | 0.22 |
| AW | 67 | M | 13 | 34 | 164.1 | 39.6 | 199 | 0.40 | 85 | 90 | 14.0 | 10.0 | 7.0 | 0.20 |
| BM | 63 | F | 16 | 36 | 195.0 | 25.9 | 272 | 0.30 | 80 | 98 | 15.0 | 14.3 | 15.0 | 0.17 |
| CJ | 57 | F | 16 | 35 | 134.0 | 20.6 | 158 | 0.27 | 95 | 98 | 9.0 | 7.0 | 10.0 | 0.08 |
| JC | 62 | F | 16 | 36 | 149.3 | 27.8 | 337 | 0.53 | 85 | 95 | 41.0 | 13.0 | 9.0 | 0.13 |
| JD | 57 | F | 17 | 33 | 138.5 | 21.5 | 145 | 0.30 | 85 | 93 | 8.4 | 13.7 | 6.0 | 0.15 |
| JN | 56 | M | 12 | 36 | 142.3 | 27.1 | 208 | 0.27 | 85 | 93 | 4.0 | 6.3 | 8.3 | 0.15 |
| LB | 66 | F | 11 | 29 | 208.4 | 32.4 | 135 | 0.63 | NA | NA | 4.2 | 6.4 | 13.0 | 0.12 |
| NG | 61 | M | 20 | 36 | 169.0 | 42.4 | 640 | 0.27 | 60 | 95 | 18.0 | 13.0 | 11.0 | 0.08 |
| NM | 56 | M | 13 | 25 | 120.5 | 21.0 | 148 | NA | 80 | 88 | 29.0 | 14.7 | 14.0 | 0.43 |
| RR | 67 | M | 19 | 35 | 138.7 | 28.2 | 486 | 2.65 | 65 | 53 | 57.6 | 13.0 | 19.0 | 0.21 |
| SB | 69 | F | 16 | 28 | 167.1 | 49.1 | 208 | 0.40 | 100 | 98 | 22.5 | 15.7 | 11.0 | 0.10 |
| SM | 64 | F | 13 | 31 | 144.8 | 17.5 | 113 | 0.33 | 73 | 90 | 52.0 | 15.7 | 11.0 | 0.19 |
| SP | 58 | M | 16 | 35 | 138.3 | 13.2 | 147 | 0.27 | 88 | 95 | 19.5 | 13.3 | 11.0 | 0.12 |
| SW | 67 | F | 13 | 34 | 154.6 | 23.6 | 249 | 0.20 | 95 | 100 | 18.0 | 10.0 | 11.0 | 0.17 |
| **Participants with aphasia** |  |  |  |  |  |  |  |  |  |  |  |  |  |  |
| AG | 62 | M | 11 | 27 | 18.0 | 7.4 | 30 | 0.93 | 65 | 85 | 34.0 | 18.8 | 16.0 | 0.28 |
| AL | 55 | F | 12 | 33 | 211.8 | 11.8 | 60 | 0.60 | 70 | 93 | 26.0 | 32.0 | 9.0 | 0.34 |
| BH | 72 | M | 11 | 24 | 50.6 | 8.2 | 38 | 0.83 | 68 | 65 | 52.0 | 19.3 | 15.0 | 0.66 |
| CH | 46 | F | 13 | 33 | 23.7 | 6.8 | 38 | 0.87 | 100 | 95 | 10.0 | 10.0 | 15.0 | 0.25 |
| DF | 51 | F | 11 | 32 | 49.6 | 10.0 | 47 | 3.80 | 65 | 88 | NA | NA | NA | 0.53 |
| DM | 56 | M | 17 | 33 | 32.6 | 6.9 | 38 | 0.17 | 73 | 85 | 60.0 | 13.3 | 16.0 | 0.14 |
| Ebo | 47 | M | 11 | 35 | 55.9 | 14.8 | 56 | 0.27 | 95 | 90 | 45.0 | 16.0 | 14.0 | 0.27 |
| GP | 63 | M | 11 | 35 | 56.3 | 11.9 | 94 | 0.80 | 80 | 85 | 24.0 | 16.0 | 20.0 | 1.31 |
| JS | 73 | F | 19 | 36 | 106.1 | 19.6 | 315 | 0.60 | 80 | 98 | 9.0 | 19.0 | 9.7 | 0.18 |
| MAD | 60 | F | 11 | 30 | 37.3 | 4.7 | 23 | 0.60 | 57 | 68 | 96.0 | 63.3 | 15.0 | 0.80 |
| MH | 69 | M | 11 | 29 | 109.9 | 9.3 | 55 | 2.43 | 83 | 90 | 70.0 | 22.7 | 13.0 | 1.11 |
| NC | 55 | M | 17 | 32 | 89.8 | 15.8 | 70 | 0.90 | 53 | 83 | 12.0 | 12.0 | 8.0 | 0.15 |
| PBL | 49 | F | 16 | 35 | 27.5 | 3.2 | 38 | 0.57 | 68 | 80 | 72.0 | NA | 18.0 | 0.25 |
| PR | 76 | F | 11 | 29 | 19.7 | 4.7 | 25 | 4.40 | 60 | 70 | 152.0 | NA | NA | 1.18 |
| RH | 68 | M | 17 | 30 | 94.4 | 19.0 | 203 | 0.33 | 95 | 98 | 13.0 | 6.3 | 10.0 | 0.33 |
| ST | 67 | F | 11 | 18 | 19.9 | 6.6 | 30 | 1.77 | 60 | 63 | 44.0 | 43.3 | 13.0 | 0.53 |
| WE | 67 | M | 10 | 33 | 55.3 | 11.6 | 69 | 2.03 | 80 | 88 | 48.0 | 27.3 | 11.8 | 0.33 |

**Supplementary Table S2: Clinical and testing data for participants with post-stroke aphasia**

| **Identifier** | **BDAE Classification** | **Months from stroke to neuropsychological testing** | **Months from stroke to psychoacoustic testing** | **Months from neuropsychological to psychoacoustic testing** |
| --- | --- | --- | --- | --- |
| **Participants with aphasia** |  |  |  |  |
| AG | Broca | 133 | 213 | 80 |
| AL | Anomia | 71 | 156 | 85 |
| BH | Mixed nonfluent | 26 | 108 | 82 |
| CH | Anomia | 38 | 72 | 34 |
| DF | Anomia | 61 | 107 | 46 |
| DM | Broca | 81 | 125 | 44 |
| Ebo | Anomia | 34 | 81 | 47 |
| GP | Anomia | 29 | 78 | 49 |
| JS | Anomia | 35 | 85 | 50 |
| MAD | Anomia | 278 | 310 | 32 |
| MH | Conduction | 13 | 41 | 28 |
| NC | Anomia | 38 | 39 | 1 |
| PBL | Conduction | 22 | 58 | 36 |
| PR | Transcortical motor | 46 | 81 | 35 |
| RH | Conduction | 14 | 36 | 22 |
| ST | Broca | 65 | 65 | 0 |
| WE | Anomia | 85 | 103 | 18 |

**Supplementary Table S3: Pure tone audiograms of participants with post-stroke aphasia and controls**

|  | **Left ear hearing level (dB)** | | | | | **Right ear hearing level (dB)** | | | | |
| --- | --- | --- | --- | --- | --- | --- | --- | --- | --- | --- |
| **Identifier** | **250Hz** | **500Hz** | **1000Hz** | **2000Hz** | **4000Hz** | **250Hz** | **500Hz** | **1000Hz** | **2000Hz** | **4000Hz** |
| **Healthy controls** |  |  |  |  |  |  |  |  |  |  |
| AB | 0 | 5 | 15 | 15 | 30 | 5 | 5 | 10 | 15 | 25 |
| ACJ | 0 | 10 | 10 | 10 | 15 | 0 | 5 | 10 | 5 | 15 |
| AT | 10 | 15 | 10 | 30 | 40 | 10 | 10 | 15 | 10 | 35 |
| AW | 15 | 30 | 10 | 15 | 35 | 20 | 20 | 15 | 10 | 35 |
| BM | 15 | 15 | 10 | 15 | 30 | 15 | 10 | 15 | 20 | 25 |
| CJ | 10 | 10 | 5 | 10 | 20 | 20 | 20 | 15 | 15 | 30 |
| JC | 15 | 20 | 10 | 20 | 35 | 15 | 15 | 15 | 10 | 15 |
| JD | 10 | 15 | 10 | 15 | 30 | 15 | 15 | 20 | 25 | 25 |
| JN | 0 | 5 | 10 | 5 | 25 | 0 | 5 | 5 | 5 | 15 |
| LB | 15 | 15 | 15 | 20 | 30 | 15 | 15 | 20 | 5 | 35 |
| NG | 15 | 15 | 15 | 15 | 30 | 35 | 15 | 10 | 15 | 30 |
| NM | 25 | 20 | 20 | 30 | 40 | 15 | 15 | 10 | 15 | 30 |
| RR | 15 | 20 | 10 | 35 | 65 | 10 | 10 | 15 | 20 | 55 |
| SB | 5 | 15 | 15 | 10 | 25 | 5 | 10 | 15 | 15 | 25 |
| SM | 10 | 15 | 10 | 25 | 45 | 10 | 15 | 10 | 30 | 40 |
| SP | 10 | 10 | 10 | 15 | 35 | 5 | 10 | 10 | 10 | 30 |
| SW | 15 | 15 | 10 | 15 | 20 | 5 | 15 | 15 | 10 | 20 |
| **Participants with aphasia** |  |  |  |  |  |  |  |  |  |  |
| AG | -10 | -5 | -5 | 15 | 30 | -5 | 5 | 10 | 20 | 45 |
| AL | 20 | 25 | 35 | 30 | 15 | 15 | 15 | 30 | 30 | 20 |
| BH | 15 | 20 | 40 | 55 | 75 | 10 | 20 | 20 | 20 | 45 |
| CH | 15 | 20 | 15 | 10 | 10 | 0 | 0 | 10 | 5 | 20 |
| DF | 30 | 30 | 30 | 50 | 65 | 15 | 15 | 10 | 25 | 25 |
| DM | 0 | 5 | 0 | 5 | 20 | 5 | 5 | 15 | 5 | 25 |
| Ebo | 10 | 10 | 5 | 10 | 10 | 20 | 10 | 5 | 5 | 15 |
| GP | -5 | -5 | 0 | 25 | 55 | 0 | 0 | 5 | 25 | 55 |
| JS | 10 | 10 | 25 | 20 | 35 | 5 | 10 | 10 | 15 | 25 |
| MAD | 0 | 10 | 20 | 40 | 60 | -5 | 5 | 20 | 45 | 50 |
| MH | -5 | -5 | 5 | 10 | 40 | 0 | 5 | 5 | 10 | 25 |
| NC | 0 | 10 | 10 | 10 | 55 | 0 | 0 | 0 | 25 | 55 |
| PBL | 0 | 10 | 10 | 5 | 15 | 5 | 10 | 10 | 5 | 5 |
| PR | 25 | 30 | 25 | 25 | 50 | 20 | 25 | 15 | 25 | 55 |
| RH | 5 | 10 | 0 | 5 | 25 | 5 | 5 | 5 | 10 | 35 |
| ST | -5 | 0 | 10 | 20 | 50 | 10 | 15 | 35 | 40 | 55 |
| WE | 0 | -5 | 10 | 55 | 60 | 10 | 5 | 5 | 25 | 55 |
| Participants are ordered alphabetically within each group. ‘dB’ = decibels; ‘Hz’ = Hertz. | | | | | | | | | | |

**Supplementary Table S4: Group level comparisons of pure tone audiogram thresholds between participants with post-stroke aphasia and controls**

| **Pure Tone Freqency (Hz)** | **Threshold in participants with aphasia (median, IQR) (dB)** | **Threshold in healthy controls (median, IQR) (dB)** | **P value** |
| --- | --- | --- | --- |
| **Left ear** |  |  |  |
| 250 | 0.0 (18.0) | 10.0 (8.0) | 0.11 |
| 500 | 10.0 (23.0) | 15.0 (8.0) | 0.13 |
| 1000 | 10.0 (23.0) | 10.0 (5.0) | 0.95 |
| 2000 | 20.0 (25.0) | 15.0 (10.0) | 0.79 |
| 4000 | 40.0 (40.0) | 30.0 (13.0) | 0.39 |
| Mean 250-1000 | 6.7 (19.2) | 11.7 (5.8) | 0.22 |
| **Right ear** |  |  |  |
| 250 | 5.0 (13.0) | 10.0 (10.0) | 0.09 |
| 500 | 5.0 (10.0) | 15.0 (5.0) | 0.08 |
| 1000 | 10.0 (13.0) | 15.0 (5.0) | 0.25 |
| 2000 | 20.0 (18.0) | 15.0 (8.0) | 0.18 |
| 4000 | 35.0 (33.0) | 30.0 (13.0) | 0.27 |
| Mean 250-1000 | 8.3 (11.7) | 11.7 (7.5) | 0.11 |
| ‘P value’ corresponds to uncorrected two-sided p-values from Mann-Whitney U-tests comparing pure tone audiometry thresholds between participants with post-stroke aphasia and healthy controls. None of the p-values are significant at the Bonferroni corrected significance threshold of p<0.004 (corrected for 12 comparisons). ‘dB’ = decibels; ‘Hz’ = Hertz; ‘IQR’ = interquartile range. | | | |

**Supplementary Table S5: Correlations between pure tone audiogram thresholds and psychoacoustic scores**

| **Psychoacoustic test** | **Left mean threshold 250-1000Hz** | **Right mean threshold 250-1000Hz** |
| --- | --- | --- |
| Pitch basic change detection threshold | 0.07 (0.80) | 0.03 (0.93) |
| Pitch detection of local change | -0.16 (0.55) | -0.24 (0.37) |
| Pitch detection of global change | 0.07 (0.80) | -0.25 (0.34) |
| Rhythm single time interval discrimination threshold | 0.03 (0.91) | 0.38 (0.15) |
| Rhythm isochrony deviation detection threshold | 0.08 (0.79) | 0.51 (0.06) |
| Rhythm metrical pattern discrimination threshold | -0.35 (0.20) | -0.12 (0.68) |
| Dynamic Modulation detection threshold | 0.03 (0.91) | 0.18 (0.48) |
| Matrix showing Spearman correlations between mean pure tone audiometry thresholds between 250-1000Hz (columns) and psychoacoustic scores (rows) for the 17 participants with post-stroke aphasia who performed psychoacoustic tests. Each cell contains the rho and uncorrected, two-sided p-value in the form: rho (p value). None of the p-values are significant at the Bonferroni corrected significance threshold of p<0.007 (corrected for 7 comparisons). ‘Hz’ = Hertz. | | |

**Supplementary Table S6: Neuropsychological test scores for the entire cohort of 76 individuals with post stroke aphasia**

| **ID** | **Nonword Repetition (30)** | **Word Repetition (80)** | **BNT (60)** | **Forward Digit Span (8)** | **CSB Word-Picture Matching (64)** | **CSB Naming (64)** | **Spoken Sentence Comprehension (32)** | **Synonym Judgement (96)** | **Raven’s (36)** | **Brixton (55)** | **BDAE ‘Cookie Theft’ description** | | | |
| --- | --- | --- | --- | --- | --- | --- | --- | --- | --- | --- | --- | --- | --- | --- |
|  |  |  |  |  |  |  |  |  |  |  | **Words Per Minute** | **Type-Token Ratio** | **Mean Length of Utterance** | **Speech Tokens** |
| AB | 8 | 62 | 17 | 3 | 61 | 34 | 24 | 72 | 32 | 49 | 56.3 | 0.52 | 16.5 | 122 |
| AD | 7 | 46 | 11 | 6 | 62 | 24 | 22 | 80 | 23 | 17 | 25.4 | 0.60 | 4.0 | 25 |
| Adr | 0 | 25 | 2 | 0 | 56 | 7 | 25 | 0 | 17 | 23 | 38.5 | 0.53 | 8.2 | 77 |
| AG | 22 | 62 | 47 | 8 | 64 | 55 | 28 | 86 | 27 | 31 | 18.0 | 0.70 | 7.4 | 30 |
| AL | 27 | 80 | 51 | 7 | 64 | 59 | 27 | 90 | 33 | 33 | 211.8 | 0.75 | 11.8 | 60 |
| AS | 0 | 28 | 0 | 2 | 49 | 4 | 16 | 41 | 17 | 26 | 18.0 | 0.33 | 2.0 | 6 |
| BH | 26 | 80 | 38 | 5 | 63 | 60 | 25 | 80 | 24 | 37 | 50.6 | 0.68 | 8.2 | 38 |
| BH | 7 | 39 | 9 | 5 | 64 | 25 | 28 | 87 | 30 | 36 | 16.7 | 0.80 | 2.9 | 25 |
| BS | 1 | 4 | 1 | 0 | 59 | 3 | 10 | 74 | 33 | 21 | 72.4 | 0.55 | 6.5 | 29 |
| CF | 10 | 56 | 7 | 2 | 57 | 19 | 22 | 74 | 33 | 28 | 8.0 | 1.00 | 1.7 | 11 |
| CH | 18 | 74 | 34 | 4 | 64 | 51 | 27 | 84 | 33 | 42 | 23.7 | 0.61 | 6.8 | 38 |
| DB | 21 | 68 | 5 | 3 | 41 | 5 | 10 | 57 | 31 | 22 | 23.9 | 0.33 | 8.8 | 120 |
| DBb | 0 | 29 | 0 | 2 | 38 | 0 | 4 | 47 | 11 | 21 | 87.2 | 0.47 | 8.2 | 32 |
| DC | 0 | 0 | 0 | 3 | 64 | 1 | 24 | 75 | 32 | 42 | 105.5 | 0.48 | 19.0 | 146 |
| DCS | 12 | 56 | 26 | 5 | 64 | 43 | 30 | 88 | 36 | 45 | 20.5 | 0.81 | 9.5 | 31 |
| DF | 16 | 75 | 28 | 3 | 64 | 49 | 20 | 75 | 32 | 24 | 49.6 | 0.72 | 10.0 | 47 |
| DL | 15 | 69 | 49 | 5 | 64 | 60 | 32 | 92 | 34 | 41 | 49.6 | 0.51 | 13.6 | 94 |
| DM | 18 | 56 | 42 | 3 | 63 | 46 | 18 | 92 | 33 | 28 | 32.6 | 0.74 | 6.9 | 38 |
| DR | 16 | 72 | 2 | 3 | 40 | 9 | 15 | 45 | 30 | 36 | 32.8 | 0.29 | 5.2 | 58 |
| DS | 17 | 71 | 45 | 4 | 64 | 52 | 28 | 90 | 26 | 40 | 47.6 | 0.87 | 13.5 | 23 |
| EB | 25 | 80 | 34 | 5 | 63 | 49 | 23 | 91 | 36 | 44 | 108.6 | 0.58 | 13.3 | 125 |
| EB | 20 | 65 | 19 | 4 | 63 | 45 | 24 | 67 | 24 | 26 | 57.0 | 0.68 | 14.8 | 56 |
| Ebo | 30 | 80 | 32 | 4 | 64 | 55 | 28 | 87 | 35 | 38 | 55.9 | 0.66 | 14.8 | 56 |
| ER | 16 | 56 | 38 | 2 | 61 | 43 | 18 | 81 | 14 | 23 | 64.2 | 0.71 | 12.8 | 63 |
| ES | 0 | 0 | 0 | 0 | 50 | 0 | 8 | 70 | 24 | 22 | 20.8 | 0.82 | 4.8 | 33 |
| Esb | 0 | 0 | 0 | 0 | 56 | 1 | 11 | 50 | 14 | 13 | 0.0 | 0.00 | 0.0 | 0 |
| GD | 0 | 4 | 0 | 2 | 50 | 10 | 16 | 47 | 23 | 19 | 1.1 | 1.00 | 0.2 | 1 |
| Gha | 24 | 79 | 43 | 4 | 61 | 52 | 30 | 92 | 30 | 38 | 47.6 | 0.55 | 16.7 | 116 |
| Gho | 5 | 50 | 7 | 2 | 54 | 17 | 14 | 44 | 22 | 19 | 7.4 | 0.67 | 4.8 | 12 |
| GL | 28 | 80 | 18 | 3 | 62 | 42 | 21 | 72 | 33 | 32 | 12.7 | 0.49 | 4.0 | 70 |
| GP | 12 | 76 | 33 | 3 | 63 | 40 | 25 | 86 | 35 | 43 | 56.3 | 0.52 | 11.9 | 94 |
| HN | 11 | 67 | 37 | 4 | 62 | 41 | 12 | 82 | 27 | 14 | 34.9 | 0.79 | 12.3 | 63 |
| JA | 21 | 68 | 37 | 3 | 64 | 53 | 25 | 61 | 29 | 34 | 63.7 | 0.65 | 8.2 | 34 |
| Jbo | 7 | 35 | 8 | 6 | 64 | 21 | 11 | 63 | 28 | 33 | 5.1 | 0.91 | 1.4 | 11 |
| JBr | 27 | 78 | 47 | 6 | 64 | 50 | 31 | 90 | 35 | 37 | 98.5 | 0.70 | 17.2 | 87 |
| JJ | 11 | 66 | 38 | 5 | 63 | 55 | 18 | 89 | 15 | 24 | 57.8 | 0.74 | 11.2 | 54 |
| JM | 25 | 80 | 38 | 4 | 64 | 53 | 32 | 79 | 34 | 42 | 109.1 | 0.68 | 11.4 | 80 |
| JM | 0 | 1 | 0 | 2 | 50 | 0 | 15 | 72 | 33 | 50 | 0.0 | 0.00 | 0.0 | 0 |
| JMf | 28 | 77 | 44 | 5 | 64 | 61 | 23 | 88 | 30 | 28 | 69.7 | 0.60 | 9.9 | 65 |
| JS | 15 | 66 | 25 | 4 | 60 | 44 | 26 | 93 | 36 | 36 | 106.1 | 0.47 | 19.6 | 315 |
| Jsa | 9 | 60 | 27 | 4 | 59 | 42 | 19 | 78 | 30 | 37 | 42.6 | 0.65 | 6.7 | 34 |
| JSb | 19 | 69 | 36 | 5 | 60 | 48 | 27 | 72 | 31 | 33 | 56.8 | 0.80 | 6.4 | 35 |
| JSc | 10 | 72 | 31 | 5 | 63 | 46 | 24 | 73 | 28 | 24 | 33.0 | 0.79 | 8.2 | 28 |
| JW | 10 | 52 | 24 | 7 | 62 | 41 | 29 | 82 | 32 | 34 | 17.2 | 0.83 | 8.0 | 18 |
| KA | 25 | 77 | 33 | 7 | 64 | 49 | 25 | 76 | 28 | 36 | 92.5 | 0.73 | 13.0 | 74 |
| KK | 10 | 45 | 5 | 0 | 60 | 26 | 15 | 78 | 36 | 42 | 20.3 | 0.54 | 6.8 | 61 |
| KL | 0 | 5 | 1 | 0 | 59 | 3 | 9 | 66 | 32 | 34 | 16.3 | 0.39 | 2.9 | 18 |
| KM | 0 | 0 | 0 | 0 | 34 | 0 | 14 | 67 | 24 | 26 | 0.0 | 0.00 | 0.0 | 0 |
| KS | 22 | 75 | 7 | 8 | 46 | 17 | 27 | 81 | 31 | 29 | 97.2 | 0.75 | 12.2 | 81 |
| KW | 0 | 3 | 0 | 4 | 61 | 0 | 27 | 79 | 29 | 28 | 2.8 | 0.75 | 1.7 | 4 |
| LH | 17 | 64 | 44 | 7 | 62 | 49 | 29 | 89 | 32 | 42 | 91.1 | 0.65 | 7.1 | 82 |
| LM | 5 | 22 | 1 | 0 | 43 | 3 | 9 | 55 | 22 | 18 | 19.9 | 0.40 | 2.5 | 10 |
| MAD | 18 | 76 | 45 | 5 | 62 | 52 | 26 | 85 | 30 | 35 | 37.3 | 0.87 | 4.7 | 23 |
| MD | 8 | 38 | 16 | 3 | 62 | 32 | 4 | 55 | 14 | 32 | 7.4 | 0.18 | 3.2 | 33 |
| MH | 0 | 11 | 3 | 2 | 64 | 4 | 19 | 67 | 29 | 41 | 109.9 | 0.66 | 9.3 | 55 |
| NC | 23 | 73 | 42 | 4 | 60 | 59 | 20 | 73 | 32 | 40 | 89.8 | 0.74 | 15.8 | 70 |
| NH | 26 | 80 | 51 | 5 | 63 | 60 | 29 | 83 | 34 | 39 | 69.5 | 0.91 | 10.3 | 22 |
| PB | 29 | 79 | 39 | 5 | 64 | 61 | 20 | 78 | 29 | 38 | 73.9 | 0.46 | 6.7 | 74 |
| PBL | 0 | 31 | 9 | 3 | 64 | 22 | 23 | 86 | 35 | 44 | 27.5 | 0.55 | 3.2 | 38 |
| PE | 4 | 36 | 6 | 2 | 62 | 13 | 16 | 76 | 29 | 23 | 67.8 | 0.53 | 12.6 | 203 |
| PM | 3 | 53 | 29 | 2 | 59 | 42 | 20 | 67 | 17 | 17 | 19.7 | 0.63 | 3.3 | 19 |
| PR | 17 | 68 | 28 | 6 | 64 | 39 | 28 | 80 | 29 | 28 | 19.7 | 0.64 | 4.7 | 25 |
| PW | 0 | 0 | 2 | 2 | 64 | 2 | 18 | 71 | 18 | 19 | 15.2 | 0.39 | 5.2 | 46 |
| Pwa | 10 | 56 | 21 | 4 | 61 | 42 | 22 | 84 | 29 | 25 | 14.6 | 0.79 | 4.8 | 19 |
| RH | 2 | 17 | 1 | 2 | 53 | 2 | 20 | 86 | 30 | 34 | 94.4 | 0.51 | 19.0 | 203 |
| RL | 4 | 49 | 32 | 5 | 62 | 55 | 20 | 89 | 29 | 40 | 59.9 | 0.78 | 8.3 | 40 |
| RR | 3 | 41 | 11 | 2 | 63 | 27 | 18 | 79 | 32 | 24 | 29.4 | 0.68 | 6.8 | 31 |
| SH | 2 | 39 | 14 | 2 | 54 | 33 | 14 | 67 | 20 | 27 | 46.6 | 0.53 | 11.7 | 73 |
| SL | 0 | 0 | 0 | 0 | 32 | 0 | 11 | 50 | 32 | 29 | 0.0 | 0.00 | 0.0 | 0 |
| ST | 15 | 64 | 30 | 2 | 59 | 51 | 23 | 85 | 18 | 21 | 19.9 | 0.67 | 6.6 | 30 |
| TA | 3 | 28 | 7 | 1 | 48 | 23 | 11 | 41 | 12 | 29 | 14.0 | 0.64 | 4.7 | 14 |
| TJ | 28 | 79 | 53 | 5 | 63 | 59 | 22 | 85 | 18 | 29 | 52.7 | 0.67 | 11.0 | 51 |
| TK | 0 | 28 | 4 | 3 | 64 | 0 | 28 | 69 | 25 | 30 | 44.0 | 0.61 | 9.5 | 66 |
| WC | 9 | 0 | 27 | 5 | 62 | 37 | 24 | 91 | 19 | 39 | 97.6 | 0.57 | 17.0 | 122 |
| WE | 14 | 59 | 31 | 5 | 64 | 51 | 27 | 84 | 33 | 39 | 55.3 | 0.59 | 11.6 | 69 |
| WM | 11 | 44 | 15 | 3 | 59 | 23 | 16 | 59 | 22 | 24 | 7.4 | 0.57 | 3.0 | 14 |
| Participants are ordered alphabetically. ‘ID’ = Participant Identifier; ‘Nonword Repetition’ = immediate non-word repetition from the Psycholinguistic Assessment of Language Processing in Aphasia subtest 8; ‘Word Repetition’ = immediate word repetition from the Psycholinguistic Assessment of Language Processing in Aphasia subtest 9; ‘BNT’ = Boston Naming Test; ‘CSB Word-Picture Matching’ = Cambridge Semantic Battery spoken word-to-picture matching; ‘CSB Naming’ = Cambridge Semantic Battery picture naming; ‘Spoken Sentence Comprehension’ = spoken sentence comprehension subtest from the Comprehensive Aphasia Test; ‘Synonym Judgement’ = 96-trial synonym judgement test; ‘Raven’s’ = Raven’s Coloured Progressive Matrices; ‘Brixton’ = Brixton Spatial Anticipation Test; ‘Words Per Minute’ = Words Per Minute on the BDAE Cookie Theft description; ‘Type-Token Ratio’ = Type-Token Ratio on the BDAE Cookie Theft description; ‘Mean Length of Utterance’ = Mean Length of Utterance on the BDAE Cookie Theft description; ‘Speech Tokens’ = number of speech tokens on the Boston Diagnostic Aphasia Examination (BDAE) Cookie Theft description’. | | | | | | | | | | | | | | |

**Supplementary Table S7: Repeated assessments of comprehension and fluency in participants with post-stroke aphasia**

| **Participant with aphasia** | **Spoken Sentence Comprehension (32)** | | | **Connected speech – Tokens** | | | | **Connected speech – Words Per Minute** | | | |
| --- | --- | --- | --- | --- | --- | --- | --- | --- | --- | --- | --- |
|  | **Assessment 1 score** | **Assessment 2 score** | **Months between timepoints** | **‘Cookie theft’ score** | **‘Discourse’ score** | **‘Narrative’ score** | **Months between timepoints** | **‘Cookie theft’ score** | **‘Discourse’ score** | **‘Narrative’ score** | **Months between timepoints** |
| **AG** | 28 | 28 | 67 | 30 | 35 | 75 | 51 | 18.0 | 23.9 | 30.6 | 51 |
| **AL** | 27 | 28 | 68 | 60 | 109 | 317 | 58 | 211.8 | 123.9 | 110.5 | 58 |
| **BH** | 25 | 23 | 69 | 38 | 59 | 69 | 59 | 50.6 | 128.3 | 76.7 | 59 |
| **CH** | 27 | 25 | 24 | 38 | 40 | 118 | 6 | 23.7 | 30.8 | 29.9 | 6 |
| **DF** | 20 | 23 | 32 | 47 | 50 | 223 | 19 | 49.6 | 47.6 | 107.7 | 19 |
| **DM** | 18 | 15 | 72 | 38 | 32 | 112 | 18 | 32.6 | 34.8 | 20.1 | 18 |
| **Ebo** | 28 | 30 | 32 | 56 | 62 | 140 | 23 | 55.9 | 88.6 | 102.2 | 23 |
| **GP** | 25 | 20 | 35 | 94 | 69 | 205 | 20 | 56.3 | 71.1 | 57.8 | 20 |
| **JS** | 26 | 20 | 34 | 315 | 225 | 383 | 22 | 106.1 | 136.4 | 116.8 | 22 |
| **MAD** | 26 | 20 | 24 | 23 | 12 | 76 | 1 | 37.3 | 60.0 | 36.5 | 1 |
| **MH** | 19 | 19 | 20 | 55 | 131 | 214 | 3 | 109.9 | 106.5 | 122.3 | 3 |
| **PBL** | 23 | 17 | 37 | 38 | 34 | 66 | 11 | 27.5 | 42.5 | 49.6 | 11 |
| **PR** | 28 | 27 | 27 | 25 | 52 | 144 | 8 | 19.7 | 29.2 | 21.8 | 8 |
| **RH** | 20 | 17 | 14 | 203 | 162 | 441 | 0 | 94.4 | 104.5 | 145.5 | 0 |
| **WE** | 27 | 28 | 10 | 69 | 190 | 202 | 0 | 55.3 | 56.2 | 59.8 | 0 |
| For the 15 participants with PSA who had more than 1 month between neuropsychological and psychoacoustic testing phases. ‘Discourse’ and ‘Narrative’ connected speech tasks were performed on the same date, at a timepoint after the ‘Cookie theft’ connected speech task. ‘Discourse’ = Procedural Discourse connected speech task; ‘Narrative’ = Storytelling Narrative connected speech task; ‘Spoken Sentence Comprehension’ = spoken sentence comprehension subtest from the Comprehensive Aphasia Test. | | | | | | | | | | | |

**Participants with PSA were stable in their cognitive performance over time**

Neuropsychological testing was conducted at least one year after the stroke, and often considerably later – i.e., the patients were either in the chronic or extremely chronic phase post onset. However, the neuropsychological and psychoacoustic testing phases occurred more than a month apart in 15 of the 17 participants with PSA (Supplementary Table S2). The issue of stability of performance in patients who are in the chronic stage after a stroke is therefore an important one. In separate projects undertaken within our group, participants have been reassessed on the spoken sentence comprehension subtest of the CAT^10^, and have undergone two more connected speech analysis tasks requiring ‘procedural discourse’ and ‘storytelling narrative’ descriptions^18^, from which the number of speech tokens and words per minute are available. Of the 15 participants with PSA who underwent neuropsychological and psychoacoustic testing more than one month apart, we found that the repeated assessment of spoken sentence comprehension correlated highly between timepoints (Spearman’s rho=0.87, p<0.0005), as did the number of speech tokens (Spearman’s rho=0.86, p<0.0005) and words per minute (Spearman’s rho=0.89, p<0.0005) produced during the ‘Cookie theft’ vs ‘Discourse’ connected speech tasks, as well as the number of speech tokens (Spearman’s rho=0.76, p=0.001) and words per minute (Spearman’s rho=0.85, p<0.0005) produced during the ‘Cookie theft’ vs ‘Narrative’ connected speech tasks. None of the 15 participants had a significant change on the spoken sentence comprehension test, which is defined in the CAT manual as a T score change of 9 or more to meet one-tailed significance of p<0.05, indicating that repeated assessments were within the range of expected test-retest variability^10^. Moreover, there is no significant correlation between the difference scores and the interval between timepoints for the CAT spoken sentence comprehension (Spearman’s rho=-0.13, p=0.66), tokens (Spearman’s rho=0.07, p=0.81) or words per minute (rho=0.21, p=0.45) produced during the ‘Cookie theft’ vs ‘Discourse’ connected speech tasks, or tokens (Spearman’s rho=-0.27, p=0.33) or words per minute (Spearman’s rho=0.06, p=0.84) produced during the ‘Cookie theft’ vs ‘Narrative’ connected speech tasks. The stability of performance for these patients over time shows that the stroke patients were stable in cognitive performance over time. In particular, the strong rank correlations between the measures of connected speech fluency over time suggests that each participant’s fluency relative to other participants within this PSA subgroup has not changed significantly over time. In keeping with this, even in the 13 participants with PSA for whom R3 data was available and who performed psychoacoustic testing more than one month after neuropsychological testing, we found that rhythm metrical pattern discrimination (R3) remained significantly, negatively correlated with all additional measures of speech fluency obtained at the second timepoint: R3 vs ‘Discourse’ tokens, Spearman’s rho= -0.72, one-sided p=0.003; R3 vs ‘Narrative’ tokens, Spearman’s rho= -0.72, one-sided p=0.003; R3 vs ‘Discourse’ words per minute, Spearman’s rho= -0.63, one-sided p=0.01; R3 vs ‘Narrative’ words per minute, Spearman’s rho= -0.73, one-sided p=0.002.

**Supplementary Table S8: Group level comparisons of speech fluency and cognitive function between participants with post-stroke aphasia and controls**

| **Neuropsychological test** | **Participants with aphasia (median, IQR)** | **Controls (median, IQR)** | **P value** |
| --- | --- | --- | --- |
| Raven’s Progressive Coloured Matrices | 32.00 (5.00) | 34.00 (6.00) | 0.53 |
| **Cookie Theft Description:** |  |  |  |
| Words Per Minute | 50.49 (66.23) | 144.26 (29.31) | 0.00003* |
| Mean Length of Utterance (morphemes) | 9.27 (6.62) | 25.00 (9.75) | 0.00002* |
| Number of speech tokens | 47.00 (35.50) | 158.00 (136.50) | 0.0004* |
| One-way rank ANCOVAs comparing neuropsychological scores between the post-stroke aphasia subgroup and controls, with years of education included as a covariate. * indicates the p-value is significant at the Bonferroni corrected significance threshold of p<0.0125 (corrected for 4 comparisons). | | | |

**Supplementary Table S9: Individual deficits of auditory processing in participants with post-stroke aphasia**

| **Participant with aphasia** | **Log_10_ pitch basic change detection threshold** | **Pitch detection of local change** | **(Pitch detection of global change)^4^** | **Log_10_ rhythm single time interval discrimination threshold** | $\boldsymbol{\surd}$**(Rhythm isochrony deviation detection threshold)** | **Rhythm metrical pattern discrimination threshold** | **Log_10_ Dynamic Modulation detection threshold** |
| --- | --- | --- | --- | --- | --- | --- | --- |
| **AG** | 0.08 | 0.04 | 0.15 | 0.11 | 0.19 | 0.12 | 0.17 |
| **AL** | 0.21 | 0.08 | 0.42 | 0.20 | 0.03 | 0.69 | 0.07 |
| **BH** | 0.11 | 0.06 | 0.02 | 0.05 | 0.17 | 0.17 | **0.006*** |
| **CH** | 0.10 | 0.81 | 0.53 | 0.64 | 0.55 | 0.17 | 0.17 |
| **DF** | **0.002*** | 0.04 | 0.22 | **NA** | **NA** | **NA** | 0.01 |
| **DM** | 0.87 | 0.19 | 0.15 | 0.09 | 0.39 | 0.13 | 0.51 |
| **Ebo** | 0.61 | 0.64 | 0.29 | 0.07 | 0.27 | 0.23 | 0.19 |
| **GP** | 0.12 | 0.24 | 0.15 | 0.20 | 0.27 | 0.02 | **0.0003*** |
| **JS** | 0.26 | 0.43 | 0.74 | 0.86 | 0.20 | 0.70 | 0.28 |
| **MAD** | 0.21 | 0.01 | 0.02 | 0.01 | **0.0005*** | 0.17 | **0.002*** |
| **MH** | **0.007*** | 0.31 | 0.29 | 0.03 | 0.11 | 0.31 | **0.0006*** |
| **NC** | 0.10 | 0.01 | 0.11 | 0.72 | 0.46 | 0.82 | 0.45 |
| **PBL** | 0.25 | 0.09 | 0.07 | 0.05 | **NA** | 0.06 | 0.12 |
| **PR** | **0.001*** | 0.02 | 0.02 | **0.004*** | **NA** | **NA** | **0.0004*** |
| **RH** | 0.56 | 0.80 | 0.73 | 0.69 | 0.78 | 0.66 | 0.04 |
| **ST** | 0.02 | 0.02 | 0.01 | 0.07 | **0.006*** | 0.31 | 0.01 |
| **WE** | 0.01 | 0.22 | 0.22 | 0.05 | 0.06 | 0.41 | 0.11 |
| Matrix showing results of Bayesian Test for a Deficit, controlling for years of education as a covariate, comparing each participant with aphasia’s (rows) performance on each of seven psychoacoustic tests (columns) to the control group. Each cell contains the uncorrected, one-sided p-value for a significance test of whether the participant with aphasia (indicated in the left-most cell of that row) has a score on the psychoacoustic test (indicated in the top-most cell of that column) that is an observation from the control group, controlling for years of education as a covariate. Participants are ordered alphabetically. Abbreviations: NA = Not Acquired as participant unable to perform this psychoacoustic task at the easiest difficulty level. * indicates the p-value is significant at the Bonferroni corrected significance threshold of p<0.007 (corrected for 7 comparisons). Cells in which participant performance was significantly impaired relative to the control group are shaded in green. | | | | | | | |

**Supplementary Table S10: Correlations between Principal Component 3 and neuropsychological tests**

| **Neuropsychological test** | **Spearman’s rho with Principal Component 3** | **P value** |
| --- | --- | --- |
| ‘Cookie Theft’ number of speech tokens | 0.92 | 0.00000006* |
| ‘Cookie Theft’ Mean Length of Utterance | 0.91 | 0.0000003* |
| ‘Cookie Theft’ Words Per Minute | 0.91 | 0.0000002* |
| Immediate non-word repetition | -0.11 | 0.34 |
| Immediate word repetition | 0.01 | 0.49 |
| Boston Naming Test | -0.24 | 0.18 |
| CSB Picture Naming | -0.06 | 0.41 |
| Forward Digit Span | -0.30 | 0.12 |
| CSB Spoken Word-to-Picture Matching | -0.33 | 0.10 |
| Spoken Sentence Comprehension | -0.30 | 0.13 |
| Synonym Judgement Test | 0.18 | 0.25 |
| ‘Cookie Theft’ Type-Token Ratio | -0.27 | 0.15 |
| Raven’s Coloured Progressive Matrices | 0.34 | 0.09 |
| Brixton Spatial Anticipation Test | 0.19 | 0.23 |
| ‘P value’ corresponds to uncorrected one-sided p-values from Spearman correlations between neuropsychological scores and Principal Component 3 score in the post-stroke aphasia subgroup who underwent psychoacoustic testing. * indicates the p-value is significant at the Bonferroni corrected significance threshold of p<0.004 (corrected for 14 comparisons). ‘CSB’ = Cambridge Semantic Battery. | | |

**Supplementary references**

1 Goodglass, H. & Kaplan, E. *The assessment of aphasia and related disorders*. 2nd ed. edn, (Lea & Febiger, 1983).

2 Jenkinson, M., Beckmann, C. F., Behrens, T. E., Woolrich, M. W. & Smith, S. M. FSL. *Neuroimage* **62**, 782-790 (2012).

3 Rorden, C. & Brett, M. Stereotaxic display of brain lesions. *Behav Neurol* **12**, 191-200 (2000).

4 Halai, A. D., Woollams, A. M. & Lambon Ralph, M. A. Using principal component analysis to capture individual differences within a unified neuropsychological model of chronic post-stroke aphasia: Revealing the unique neural correlates of speech fluency, phonology and semantics. *Cortex* **86** (2017).

5 Butler, R. A., Lambon Ralph, M. A. & Woollams, A. M. Capturing multidimensionality in stroke aphasia: mapping principal behavioural components to neural structures. *Brain* **137**, 3248-3266 (2014).

6 Kay, J., Lesser, R. & Coltheart, M. *PALPA: psycholinguistic assessments of language processing in aphasia.*, (Erlbaum, 1992).

7 Kaplan, E., Goodglass, H. & Weintraub, S. *Boston naming test*. (Lea & Febiger, 1983).

8 Wechsler, D. A. *Wechsler memory scale—revised manual*. (Psychological Corporation, 1987).

9 Bozeat, S., Lambon Ralph, M. A., Patterson, K., Garrard, P. & Hodges, J. R. Non-verbal semantic impairment in semantic dementia. *Neuropsychologia* **38**, 1207-1215 (2000).

10 Swinburn, K., Baker, G. & Howard, D. *CAT: the comprehensive aphasia test*. (Psychology Press, 2005).

11 Jefferies, E., Patterson, K., Jones, R. W. & Lambon Ralph, M. A. Comprehension of concrete and abstract words in semantic dementia. *Neuropsychology* **23**, 492-499 (2009).

12 Raven, J. C. *Advanced progressive matrices, set II*. (H. K. Lewis, 1962).

13 Burgess, P. W. & Shallice, T. *The Hayling and Brixton tests*. (Thames Valley Test Company, 1997).

14 Quade, D. Rank analysis of covariance. *Journal of the American Statistical Association* **62**, 1187-1200 (1967).

15 Crawford, J. R., Garthwaite, P. H. & Ryan, K. Comparing a single case to a control sample: testing for neuropsychological deficits and dissociations in the presence of covariates. *Cortex* **47**, 1166-1178 (2011).

16 Crawford, J. R. & Garthwaite, P. H. Methods of testing for a deficit in single-case studies: Evaluation of statistical power by Monte Carlo simulation. *Cogn Neuropsychol* **23**, 877-904 (2006).

17 Preacher, K. J. & MacCallum, R. C. Exploratory factor analysis in behavior genetics research: factor recovery with small sample sizes. *Behav Genet* **32**, 153-161 (2002).

18 Alyahya, R., Halai, A., Conroy, P. & Lambon Ralph, M. A Unified Model of Post-Stroke Language Deficits Including Discourse Production and Their Neural Correlates. *Brain* (2020).
